# Adherence to the bedside paediatric early warning system (BedsidePEWS) in a pediatric tertiary care hospital

**DOI:** 10.1186/s12913-021-06809-2

**Published:** 2021-08-21

**Authors:** Orsola Gawronski, Federico Ferro, Corrado Cecchetti, Marta Ciofi Degli Atti, Immacolata Dall’Oglio, Emanuela Tiozzo, Massimiliano Raponi

**Affiliations:** 1 Professional Development, Continuing Education and Research Unit, Bambino Gesù Children’s Hospital IRCCS, P.zza S. Onofrio 4, 00165 Rome, Italy; 2 Pediatric Intensive Care Unit, Department of Emergency, Acceptance and General Pediatrics, Bambino Gesù Children’s Hospital IRCCS, P.zza S. Onofrio 4, 00165 Rome, Italy; 3 Clinical Epidemiology Unit, Bambino Gesù Children’s Hospital IRCCS, P.zza S. Onofrio 4, 00165 Rome, Italy; 4 Medical Directorate, Bambino Gesù Children’s Hospital IRCCS, P.zza S. Onofrio 4, 00165 Rome, Italy

**Keywords:** Monitoring, Resuscitation, Early warning score, Track and trigger system, Deteriorating children, Chronic illness, Acute illness, Pediatric

## Abstract

**Background:**

The aim of this study is to describe the adherence to the Bedside Pediatric Early Warning System (BedsidePEWS) escalation protocol in children admitted to hospital wards in a large tertiary care children’s hospital in Italy.

**Methods:**

This is a retrospective observational chart review. Data on the frequency and accuracy of BedsidePEWS score calculations, escalation of patient observations, monitoring and medical reviews were recorded.

Two research nurses performed weekly visits to the hospital wards to collect data on BedsidePEWS scores, medical reviews, type of monitoring and vital signs recorded. Data were described through means or medians according to the distribution. Inferences were calculated either with Chi-square, Student’s t test or Wilcoxon-Mann–Whitney test, as appropriate (*P* < 0.05 considered as significant).

**Results:**

A total of 522 Vital Signs (VS) and score calculations [BedsidePEWS documentation events, (DE)] on 177 patient clinical records were observed from 13 hospital inpatient wards. Frequency of BedsidePEWS DE occurred < 3 times per day in 33 % of the observations. Adherence to the BedsidePEWS documentation frequency according to the hospital protocol was observed in 54 % of all patients; in children with chronic health conditions (CHC) it was significantly lower than children admitted for acute medical conditions (47 % vs. 69 %, *P* = 0.006). The BedsidePEWS score was correctly calculated and documented in 84 % of the BedsidePEWS DE. Patients in a 0–2 BedsidePEWS score range were all reviewed at least once a day by a physician. Only 50 % of the patients in the 5–6 score range were reviewed within 4 h and 42 % of the patients with a score ≥ 7 within 2 h.

**Conclusions:**

Escalation of patient observations, monitoring and medical reviews matching the BedsidePEWS is still suboptimal. Children with CHC are at higher risk of lower compliance. Impact of adherence to predefined response algorithms on patient outcomes should be further explored.

## Background

Clinical deterioration in children admitted to hospital wards is often detected through signs of increasing severity of illness, which otherwise may lead to unplanned Pediatric Intensive Care Unit (PICU) admissions or cardiac arrest [1, 2]. Pediatric Early Warning Systems (PEWS) have been devised to detect signs of clinical instability with the purpose to activate appropriate and timely interventions to prevent evolving into critical illness. The timely activation of a response is usually recommended in trigger systems when one or more of the thresholds for abnormal observations are breached. Alternatively the magnitude of deviation from normal observations is synthetized in a score, as in scoring systems [3]. In a case control study that evaluates the performance of 18 PEWS, scoring systems performed better than trigger systems, reaching higher specificity and Area Under Roc Curve (AUROC) values than trigger systems. The Bedside Pediatric Early Warning System (BedsidePEWS) ranked second, with an AUROC of 0.88 (CI 0.85 to 0.91) [4].

The BedsidePEWS was developed by Parshuram et al. [5] who conducted a multicenter evaluation of its performance and the first test of its effect on significant patient outcomes through a clustered randomized controlled trial [6]. BedsidePEWS performance was reported to be more effective than the retrospective nurses’ ratings of deteriorating patients urgently admitted to the PICU [5]. In the Evaluation of Processes of care and Outcomes of Children in Hospital (EPOCH) trial, the BedsidePEWS enabled to significantly reduce the severity of illness of patients urgently admitted to the PICU, but had no significant effect on child mortality [6]. Some evidence of PEWS effectiveness was reported in a systematic review where PEWS were part of a wider systems approach involving a Rapid Response Team (RRT) or Medical Emergency Team (MET) ready to respond to deteriorating patients, with a potential to reduce 31 deaths/10.000 hospital admissions [7]. However, evidence is limited since most studies are single-centered and mainly performed in specialist hospitals [8].

Studies on PEWS implementation have shown an increase in VS documentation on patient charts when a PEWS is used [6]. However incorrect PEWS age charts and score calculation errors are still frequent when paper documentation is used [7, 9, 10]. Moreover, significant variation in adherence to guidelines on VS assessments and recommended actions for deteriorating patients have been reported [11]. More research is still required to describe compliance to PEWS response algorithms and process outcomes, such as the frequency and type of vital signs monitored, the frequency of score documentation and physician reviews. Moreover, adherence to the PEWS response algorithms in admitted children and young adults with chronic conditions may be substantially different because of their basal altered vital signs thresholds. On the other hand, this increasing population of children is at a higher risk of severe acute illness and unplanned PICU admissions than other pediatric patients [12, 13]. Therefore, a 6-month observational audit was performed to assess the use of the BedsidePEWS and escalation practices in children with acute and chronic illnesses on the wards of a tertiary care pediatric hospital in 2018.

## Methods

This is a retrospective chart review. The aim of this audit was to describe compliance with the use of the BedsidePEWS in a large tertiary care children’s hospital: (1) Use of the correct chart by age group according to the patient’s age; (2) Frequency and type of vital signs, monitoring and documented physician reviews in all children, children with high BedsidePEWS scores (scores ≥ 7 and ≥ 5 < 7) and children with acute and chronic health conditions (CHCs). CHCs are defined as illnesses that last for 3 months or more or require long term care [14, 15]; (3) Presence and adequacy of score calculation. The audit was performed from July to December 2018 by two research nurses, members of the research team and BedsidePEWS experts. A convenience sample of 10 BedsidePEWS charts in every ward, if available at the time of the audit was selected, to collect data regarding the last 24 h of admission. Patients included in this audit were admitted to the ward for ≥24 h and had an age < 18 years. Children admitted to intensive care units were excluded. The data sources were the BedsidePEWS charts and the medical records. Data were collected on a paper pro forma and then entered into an Excel database. Data were checked for consistency and accuracy by a second nurse who was a member of the research team. Each set of at least 5 BedsidePEWS clinical indicators and BedsidePEWS score was defined as a BedsidePEWS documentation event (DE) for the purpose of this audit. The vital signs observed were the BedsidePEWS clinical indicators [heart rate (HR), respiratory rate (RR), systolic blood pressure (SBP), oxygen (O), transcutaneous oxygen saturation (SpO2), capillary refill time (CRT) and work of breathing (WoB)], as well as level of consciousness (LoC) and temperature (T). Any missing vital signs were assumed to be normal. Each BedsidePEWS score was manually recalculated by the research nurse to check for calculation errors. Data on medical reviews, type of monitoring [Electrocardiogram (ECG), SpO2 monitoring or intermittent] and patient characteristics (age, gender, disease, type of ward, acute or chronic condition) were also collected from the clinical record. Staff were informed of the study by their ward nurse manager. The data were collected once a week, excluding weekends. Theresearch team was composed of a PICU physician, a quality improvement expert, two research nurses and the BedsidePEWS implementation coordinator. Optimal frequency of VS/BedsidePEWS monitoring and documented medical reviews was assessed according to the compliance with local timing thresholds by BedsidePEWS score range as described on Table 1.

**Table 1:** Timing audited for Vital Signs/BedsidePEWS documentation and medical review by BedsidePEWS score range

BedsidePEWS score range	VS/ BedsidePEWS score timing (hours)	Medical review, timing (hours)
0-2	≤8	≤24
3-4	≤4	≤6
5-6	≤2	≤4
≥7	≤1	≤2

### Setting

The audit was performed in a large tertiary care pediatric hospital in Central Italy. The hospital has a total of 607 beds across two sites, 25 hospital wards and 4 PICUs/Cardiac Intensive Care Units (CICU) and 1 Neonatal Intensive Care Unit (NICU), with a total of 465 beds at the main site where the audit for the present study was performed. There are also 4 High Dependency Units (HDU) for cardiac, respiratory and hematology-oncology patients with a total of 77 beds for non-invasive ventilation (NIV), inotropic support and isolation if required. The hospital provides care for the pediatric population of the central and southern regions of Italy. The total number of admissions in 2018 was 19,878. Nurse/patient ratios ranged from 1/4 in HDUs to 1/10 on pediatric wards. Vital sign monitoring was performed by staff nurses on the wards through portable or bedside monitoring systems and patient observation. Medical reviews were performed by the ward or department consultants.

### The BedsidePEWS

The BedsidePEWS was first introduced in 2014. A daily hard copy paper, age specific, BedsidePEWS chart was customized to document vital signs and calculate the score. A color-code system was used to define score thresholds and rapidly identify any variance of vital signs from normal values, according to five different age groups (< 3 months, 4–12 months, > 1 < 4 years, 4–12 years, > 12 years). The clinical indicators of the BedsidePEWS, scores and sub-scores are presented in Table 2.

**Table 2:** The Bedside Pediatric Early Warning System clinical indicators and subscores

Clinical indicator	Subscores (points)
Heart rate (beats/minute), points	Deviation from normal ranges (0: normal value; 1: mild deviation; 2: moderate deviation; 4: severe deviation) by age group (0-3 months, 3-12 months, 1-4 years, 4-12 years, >12 years)
Systolic blood pressure (mmHg)	
Respiratory rate (breaths/minute)	
Respiratory effort	0: normal; 1: mild; 2: moderate; 4: severe/any apnea
Oxygen saturation (%)	0: >94%; 2: ≤90%
Oxygen therapy	0: room air; 2: oxygen therapy <4 l/minute or <50%; 4: oxygen therapy ≥4 l/ minute or ≥ 50%
Capillary refill time	0: <3 seconds; 4: ≥ 3seconds

Note: adapted from Parshuram C. S. et al, [5]

Other vital signs which are not included among the BedsidePEWS clinical indicators, such as temperature (T) and level of consciousness (LoC) are also required by the hospital Vital Signs (VS) protocol. A score matched response algorithm was embedded in the chart to define: (1) score calculation and VS documentation frequency; (2) timing for medical and nursing review; (3) recommended distribution of high score patients among the nursing team; and, (4) type of monitoring (continuous cardiac monitoring, SpO2 or intermittent) [6, 16]. A Vital Signs (VS) protocol with the embedded BedsidePEWS was edited. The documentation of a minimum of 5 clinical indicators of the BedsidePEWS is mandatory as it is required to calculate the score with a sufficient discriminating power between children who were very sick and those who were in better conditions [17] Any missing scores were assumed to be normal. The RRT/MET is called at a score of 7 or more. The BedsidePEWS was integrated into the handover hospital protocol to improve communication on high-risk children on the wards and between healthcare professionals and the RRT/MET. The score was also validated at the hospital’s Bone Marrow Transplant Unit showing a good screening performance in this high-risk patient population [17]. The importance of using clinical judgement over the BedsidePEWS score was highlighted on the VS protocol and discussed for specific patient populations with respiratory or cardiac chronic diseases, where the score trend is more relevant than the absolute score to detect clinical deterioration.

### Analysis

Data were summarized as proportions for categorical variables and as means or medians for continuous variables as appropriate according to the distribution, tested with the Kolmogorov–Smirnov test. The proportion of BedsidePEWS DE compliant with the expected frequency or type of monitoring, by BedsidePEWS score range was calculated for the highest BedsidePEWS scores found on the patient charts during the previous 24-hours. The proportion of documented medical reviews compliant with the recommended timing was calculated for all the BedsidePEWS DE recorded for the study group. The timing for the BedsidePEWS re-assessments, medical reviews and type of monitoring by BedsidePEWS score range, patient characteristics, hospital setting and highest BedsidePEWS score, were compared among patients with acute and chronic conditions. Inferences were calculated with chi-square, Student’s t test or Wilcoxon-Mann–Whitney test, as appropriate (a p-value < 0.05 is considered statistically significant). The statistical package STATA version 15.0 was used for analysis (StataCorp LP, College Station, TX). The study was approved by the hospital research board and was exempt from consent.

## Results

**Fig. 1:**
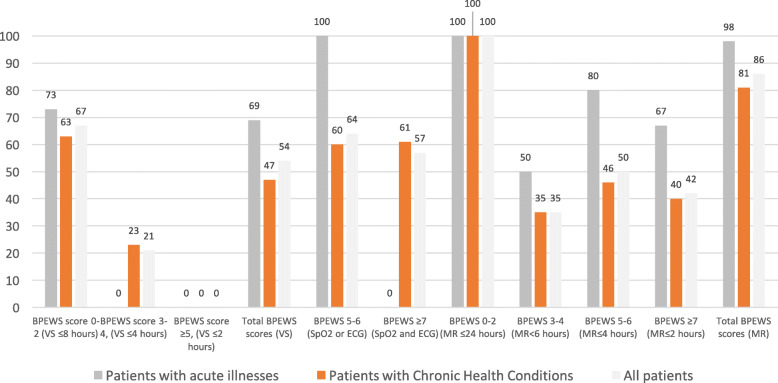
Adherence to the BedsidePEWS. The proportion (%) of patients with BedsidePEWS documentation events according to predefined criteria/audit thresholds (time interval and type of monitoring, by score range) was calculated for the highest BPEWS scores found on the clinical record during the 24-h observation period, presented in Table 3. Adherence to recommended timing for medical reviews was calculated over all BedsidePEWS scores (522) recorded for the study group. Legend: BPEWS, BedsidePEWS; ECG, Electrocardiogram monitoring; SpO2, percutaneous blood oxygen saturation VS, vital signs; MR, medical review

During the study period, a total of 522 BedsidePEWS DE on 177 patient clinical records were observed from 13 wards (4 Pediatric Specialty Units, 4 High Dependency Units, 3 Surgical Pediatric wards, and 2 General Medical Pediatric wards). Children with chronic health conditions (CHC) were 122 (69 % of the observed patients), and 25 (14 %) children had signs of physiological instability with a BedsidePEWS score ≥5. Median BedsidePEWS scores were significantly different between children with acute illnesses (median BedsidePEWS = 0, IQR = 0–0) and CHC (median BedsidePEWS = 1, IQR = 0–2, *P* < 0.001). The patient characteristics are shown in Table 3. The appropriate age specific chart was used in 99 % of the observations. BedsidePEWS DE were present on patient charts with a mean frequency of 3.34 times (CI 3.11–3.57) in 24 h. The frequency of BedsidePEWS DE was below the minimum required hospital standard (every 8 h, 3 times a day) in 33 % (*n* = 85) of the patients. Adherence to BedsidePEWS documentation frequency according to the hospital protocol and audit thresholds was observed in 54 % (*n* = 95) of the patients and was lower than 50 % (43 %; *n* = 37) in the hematology-oncology, cardiology and other specialty wards. BedsidePEWS DE frequency in patients with CHC was significantly lower than in patients with acute medical conditions admitted to a hospital ward (47 % vs. 69 %, *P* = 0.006). When the BedsidePEWS score was > 4, a BedsidePEWS DE was performed in over 2 h. When considering the 522 BedsidePEWS DE in the study group (177 patients), adherence to the medical review (MR) recommendations was 86 %, significantly higher in the acute patient group compared to children with CHC (158 MR in acute patients (98 %) compared to 291 (81 %) in children with CHC, *P* < 0.001). Adherence to the timing of BedsidePEWS DE, medical reviews and type of monitoring by BedsidePEWS score ranges are shown in Fig. 1.

**Table 3:** Patient characteristics

	Children with Acute illnesses	Children with CHC	Total	*P*-value
Characteristic				
No. of patients	55	122	177	
Age (years, mean±SD)	6.4±5.58	6.5 ±5.6	6.47±5.56	.91
Age, No. (%)				.09
Age <1 year	4 (7)	1 (1)	5 (2)	
Age 1-4 years	22 (40)	59 (48)	81 (46)	
Age 5-11 years	15 (27)	34 (28)	49 (28)	
Age ≥12 years	14 (25)	28 (23)	42 (24)	
Sex (female), No. (%)	28 (51)	51 (42)	79 (45)	.26
Disease, No. (%)				**<.001**
Respiratory	10 (18)	5 (4)	15 (8)	
Cardiovascular	0 (0)	26 (21)	26 (15)	
Endocrine-metabolic	5 (9)	12 (10)	17 (10)	
Genetic syndromes	0 (0)	8 (7)	8 (4)	
Surgical	9 (16)	5 (4)	14 (8)	
Infectious	11 (20)	7 (6)	18 (10)	
Skeletal-muscle diseases	13 (24)	8 (7)	21 (12)	
Neurological	4 (7)	23 (19)	27 (15)	
Haematological, Oncological	0 (0)	22 (18)	22 (12)	
Renal	3 (5)	6 (5)	9 (5)	
Ward, No. (%)				**<.001**
Medical Ward	15 (27)	10 (8)	25 (14)	
Specialty Ward	6 (11)	55 (45)	61 (34)	
Surgical Ward	13 (24)	13 (11)	26 (15)	
High Dependency Unit	21 (38)	44 (36)	65 (37)	
Highest BPEWS^a^, No. (%)				**.005**
BPEWS 0-2	52 (94)	86 (70)	138 (78)	
BPEWS 3-4	1 (2)	13 (11)	14 (8)	
BPEWS 5-6	1 (2)	10 (8)	11 (6)	
BPEWS ≥7	1 (2)	13 (11)	14 (8)	

Abbreviations: *BPEWS* BedsidePEWS, *CHC* Chronic Health Conditions, *SD* Standard Deviation

^a^This was the highest BedsidePEWS score found on the patients’ clinical records during the 24-h observation period

Of the 522 BedsidePEWS DE in 99 % (*n* = 520) ≥5 clinical indicators of the BedsidePEWS were documented but all of the 7 clinical indicators were documented in 93 % (*n* = 482) of the BedsidePEWS DE. The BedsidePEWS score was correctly calculated and documented in 84 % (*n* = 439) of the observed BedsidePEWS DE. Of the 522 score calculations, in 56 (11 %) the score was wrongly calculated, and in 27 (5 %) no score was documented. In > 95 % of the observations, heart rate, respiratory rate, transcutaneous oxygen saturation, oxygen therapy, capillary refill time, work of breathing and level of consciousness were documented. Temperature was recorded in 79 % (*n* = 410) and systolic blood pressure (SBP) in 89 % (*n* = 465) of the BedsidePEWS DE. SBP documentation was significantly higher in patients with CHC compared to children with acute illnesses (94 % vs. 78 %, *p* < 0.001), while Temperature documentation was significantly lower in children with CHC (76 % vs. 84 %, *p* = 0.023).

## Discussion

This study explored BedsidePEWS monitoring and physician review escalation practices matched with BedsidePEWS scores in a large tertiary care pediatric hospital. We found that BedsidePEWS monitoring was still suboptimal (≥ 4 hourly) in patients at risk of clinical deterioration with a BedsidePEWS > 4. This result might be reflective of the possibility of applying nursing clinical judgement to establish the timing for BedsidePEWS re-evaluations foreseen in our hospital VS and BedsidePEWS protocol. In other contexts nurses have been found to use PEWS scores using their own clinical judgement through a process of intuition based on experience, to confirm their assessment of evolving critical illness rather than using solely the score [18]. Moreover, repeated exposure to children with chronic illnesses, prevalent in this audit in the higher BedsidePEWS group, might have determined a ≥ 4 hourly frequency of documented patient observations possibly based on these children’s abnormal baseline VS thresholds. Other factors, such as staffing, workload, skill mix, access to competency based education and ward cultures should be further investigated in relation to their potential impact on our hospital’s PEWS escalation practices [19–22].

We found that compliance with the BedsidePEWS monitoring timing thresholds was higher in the group of patients admitted for acute clinical conditions (*n* = 38/55 complying patients, 69 %) compared to children with chronic illnesses (*n* = 57/122 complying patients, 47 %), which constituted the majority of our observations (122, 69 %). Patients with chronic diseases, such as congenital heart disease, metabolic diseases or neurological impairment may have altered basal vital signs and higher thresholds due to their condition. BedsidePEWS high scores have been reported in these patients, particularly in relation to increasing risk factors such as medical or organizational complexity, the use of medical devices or recent transitions from other wards [5]. Score trends rather than PEWS absolute values might be more reflective of increasing severity of illness to determine VS monitoring plans [23]. However, proximity to PICU admission, up to 12 h, has been found to increase with BedsidePEWS scores > 6, making this a population of children at increased risk of clinical deterioration [24]. Patients with chronic illnesses constitute an increasing proportion of the total number of pediatric hospital admissions with a higher technological dependency on digital devices, increased risk of PICU readmissions, hospital length of stay and mortality [25–27]. The impact of the use of BedsidePEWS on unplanned PICU admissions of this vulnerable patient group needs to be further explored.

Patient review from a physician is another essential element of escalation of care in relation to BedsidePEWS scores. Less than 50 % of the patients with a BedsidePEWS≥3 had a documented review by a physician according to the audit timing thresholds by the BedsidePEWS score range. This may be due to several factors. First, considering the elevated proportion of children with CHC in this audit, medical reviews might have been deferred in these children if the need for oxygen, ventilation, reduced SpO2 or tissue perfusion were altered but stable and acceptable according to the basal chronic condition of the child. Second, other factors such as low situational awareness for deteriorating patients, workload and production pressure, low nursing empowerment to call for help, and internal hierarchies slowing down the referral process might have contributed to this result [22, 28]. Third, the audit monitored all medical reviews written on the patient medical record; medical reviews that were performed but not recorded were not accounted for, with the potential risk of underestimating the frequency of medical evaluations completed on patients at higher risk. Other staffing layers of intervention including charge nurses, clinical nurse specialists, medical fellows or residents may be considered in this and similar contexts to support the timeliness of documented patient reviews.

The score was correctly calculated in 84 % (*n* = 438) of the observed BedsidePEWS DE, 5 % (*n* = 27) were missing, and the rest were incorrect (*n* = 56, 11 %). Missing scores, calculation errors and delay in patient referral have been previously reported to be possibly influenced by low situational awareness and other factors affecting decision making [18, 29]. PEWS calculation errors rate up to 17.5 % and missing scores have been described [30]. Scoring errors have been reported to be significantly higher in patients urgently admitted to PICU or deaths on the wards [7]. Scoring errors and underscoring often occur during the initial phase of clinical deterioration. The underscoring of deteriorating patients has often been found to reflect nurses’ perceptions of patient’s risk rather than being the result of a calculation error [31]. In some cases, nurses reported that they may underestimate the vital sign rather than record an abnormal observation that may require, by protocol, an escalation of care [32]. Moreover, errors were reported much more likely in observation sets where some vital signs were missing, or at the onset of clinical deterioration, reducing the opportunity to achieve early detection and appropriate interventions [33]. The advent and use of electronic medical records (EMRs) will overcome this issue by providing an electronic registration of VS and score calculations, possibly also giving prompts to recall VS and PEWS assessments [30].

In 7 % (*N* = 37) of the BedsidePEWS DE, not all of the seven clinical indicators of the BedsidePEWS were recorded. This might have underestimated the BedsidePEWS score in those patients, possibly determining a lower escalation response compared to the patient’s severity of illness. However, 99 % of the BedsidePEWS DE had at least 5 clinical indicators, sufficient to calculate a valid BedsidePEWS score. The lowest rates of recorded VS were found for Temperature (*n* = 412, 79 %), SBP (*n* = 464, 89 %) and LoC (*n* = 501, 96 %). This trend is similar, but the rates are higher than the ones found in another recent study describing patient observations on pediatric wards [7].

This study has some limitations. Firstly, it is a single-center study, possibly limiting the generalizability of the findings to other settings. Secondly, the convenience sample of the clinical records available on the audit day may have introduced a selection bias, due to the possible unavailability of clinical records of patients temporarily out of the ward for diagnostic or therapeutic procedures at the time of data collection. Thirdly, most BedsidePEWS scores reported in this study were low, limiting the possibility of describing with greater precision the adherence to the BedsidePEWS in patients at higher risk. Moreover, the BedsidePEWS and medical review information recorded on the clinical record might not be fully reflective of healthcare providers’ patient observations and reviews performed for higher risk patients. Strategies to increase BedsidePEWS monitoring and the documentation of medical reviews, including the implementation of the Electronic Medical Record (EMR) adopted by the hospital in 2019, with appropriate BedsidePEWS scoring and MR prompts, are recommended to increase the completeness and timeliness of escalation of care according to the BedsidePEWS.

## Conclusions

This audit describes the use of the BedsidePEWS in a large tertiary care children’s hospital. Routine BedsidePEWS monitoring was performed according to the required standard in 67 % of the audited patients. Children at higher risk with a BedsidePEWS > 4 did not receive more frequent monitoring than ≥ 4 hourly. In addition, BedsidePEWS monitoring and medical reviews were less frequent in patients with chronic medical conditions. These children should be closely reviewed for changing trends of the BedsidePEWS clinical indicators as they might be reflective of impending clinical deterioration events.

The clinical relevance of these data needs to be further analyzed by looking at patient outcomes related to the completeness and frequency of patient observations, scoring accuracy and reviews. The impact of timely compared to delayed escalation of care on PICU urgent admissions and the role of the EMR in supporting this process deserves further study. Organizational issues and human factors, such as staffing, access to and availability of clinical data and team-based processes of care should be explored to support patient observation and the early recognition of escalation of care.

## Data Availability

The datasets used and/or analysed during the current study are available from the corresponding author on reasonable request.

## Abbreviations

VS: Vital signs
CHC: Chronic health conditions
PEWS: Pediatric Early Warning System
PICU: Pediatric Intensive Care Unit
CICU: Cardiac Intensive Care Unit
NICU: Neonatal Intensive Care Unit
HDU: High Dependency Unit
AUROC: Area Under Roc Curve
EPOCH: Evaluation of Processes of care and Outcomes of Children in Hospital trial
BedsidePEWS: Bedside Pediatric Early Warning System
RRT: Rapid Response Team
MET: Medical Emergency Team
MR: Medical review
NIV: Non-invasive ventilation
HR: Heart rate
RR: Respiratory rate
SBP: Systolic blood pressure
RE: Respiratory effort
CRT: Capillary refill time
O: Oxygen therapy
SpO2: Percutaneous oxygen saturation
T: Temperature
LoC: Level of consciousness
ECG: Electrocardiogram monitoring
EMR: Electronic medical record
DE: Documentation events
